# Floating Thrombus as an Underdiagnosed Etiology for Stroke: A Case Series

**DOI:** 10.7759/cureus.103909

**Published:** 2026-02-19

**Authors:** Anchal Aggarwal, Rajeshwar Sahonta, Vineeth Jaison, Jeyaraj D Pandian

**Affiliations:** 1 Department of Neurology, Christian Medical College and Hospital, Ludhiana, IND

**Keywords:** anticoagulation, carotid arteries, free-floating thrombus, intraluminal thrombus, ischemic stroke

## Abstract

Free-floating thrombus or intraluminal thrombus (FFT/ILT) of the cervicocephalic arteries is an important but underrecognized cause of ischemic stroke with high embolic risk. Here, we describe the varied clinical, radiological, etiological, and management strategies in patients with FFT. Five patients with acute ischemic stroke and FFT in the cervicocephalic arteries were identified by computed tomography angiography after excluding other causes. The patients received individualized treatment, with follow-up imaging and clinical evaluation. FFT occurred in the aortic arch and carotid bifurcation. Etiologies included atherosclerosis and prothrombotic states such as systemic lupus erythematosus. Two patients underwent thrombolysis without deterioration. All received antithrombotic therapy and statins, showing thrombus resolution or reduction with neurological improvement. FFT is an emerging, underrecognized cause of stroke. Early imaging diagnosis and individualized treatment yielded favorable outcomes, but established guidelines are still lacking, highlighting the need for larger studies.

## Introduction

Free-floating thrombus (FFT)/intraluminal thrombus (ILT) refers to a friable thrombus that is partially attached to the vessel wall with one end freely mobile within the arterial lumen. It exhibits a pendulous motion synchronized with each cardiac cycle, which predisposes it to fragmentation and distal embolization, potentially resulting in acute ischemic stroke (AIS). FFT formation is often associated with underlying vessel wall abnormalities, including ulcerated atherosclerotic plaques, arterial dissection, trauma, fibromuscular dysplasia, and vasculitis. Systemic prothrombotic conditions, such as malignancy, pregnancy, antiphospholipid antibody syndrome, inherited thrombophilia, and sepsis, may further contribute to its development. FFTs are most reported in the carotid arteries with an estimated incidence of 0.05%-1.45% among patients presenting with ischemic stroke. Owing to their unstable nature and embolic potential, early identification and appropriate management are critical to prevent recurrent or catastrophic cerebrovascular events [1].

Various treatment modalities have been tried to tackle ischemic stroke, but it is important to recognize ILTs in time. First, emergency open surgical revascularization, particularly in cases where the thrombus extends distally, carries a significant risk of thrombus dislodgement and distal embolization. Second, the use of intravenous thrombolysis (IVT) with tissue plasminogen activator (tPA) remains risky due to concerns about thrombus fragmentation and the potential for recurrent embolic events. Third, endovascular aspiration techniques have emerged as a promising minimally invasive alternative for direct thrombus removal, though their efficacy and safety require validation in larger, controlled studies [2].

While most symptomatic carotid artery FFTs are attributed to the rupture of underlying atherosclerotic plaques and commonly coexist with significant carotid stenosis, non-atherosclerotic mechanisms must also be considered, which require evaluation for cardioembolic sources, hypercoagulability, and malignancy. The optimal management of FFT in the setting of significant carotid stenosis, whether immediate revascularization or medical therapy, continues to be debated [3].

Finally, several small observational series have reported favorable outcomes with short-term anticoagulation using intravenous unfractionated heparin or low-molecular-weight heparin (LMWH), suggesting that this approach may facilitate thrombus resolution while minimizing embolic risk [2]. In terms of natural history, FFT may involve progression to complete arterial occlusion, distal embolization, or stabilization. Despite this, the optimal management strategy remains uncertain, particularly with respect to the selection and duration of antithrombotic therapy, the potential risks and benefits of IVT, and the appropriate timing and efficacy of interventional treatments such as carotid artery stenting or carotid endarterectomy [4].

Current evidence is limited to isolated case reports and small observational studies, with no randomized controlled trials or large prospective cohorts available to guide treatment decisions. Therefore, there is still a need for further studies to establish evidence-based management guidelines for FFT [2].

In the Indian context, most reported cases of FFT have emerged during the COVID-19 pandemic, reflecting the prothrombotic state associated with coronavirus infection and its vascular complications. However, data on FFT unrelated to COVID-19 infection remain limited. Here, we present a case series of five patients diagnosed with ILT at a single tertiary care center who are unrelated to the COVID-19 pandemic. This case series highlights the need for larger prospective studies, particularly in the Indian setting, to better elucidate the underlying etiological factors contributing to the development of floating thrombus and to establish evidence-based treatment guidelines aimed at preventing stroke recurrence and reducing the overall burden of stroke. This also highlights the need for prompt recognition and appropriate management guidelines before surgical or endovascular interventions for stroke to prevent thrombus dislodgement and further embolization.

## Case presentation

Case 1

A 63-year-old male, a known hypertensive (not on regular medication) and chronic smoker, presented to the emergency department with sudden-onset left-sided weakness; he arrived approximately 11 hours after symptom onset.

On neurological examination, he had left face, arm, and leg weakness with hemisensory impairment. The National Institutes of Health Stroke Scale (NIHSS) score at presentation was 17.

**Figure 1:**
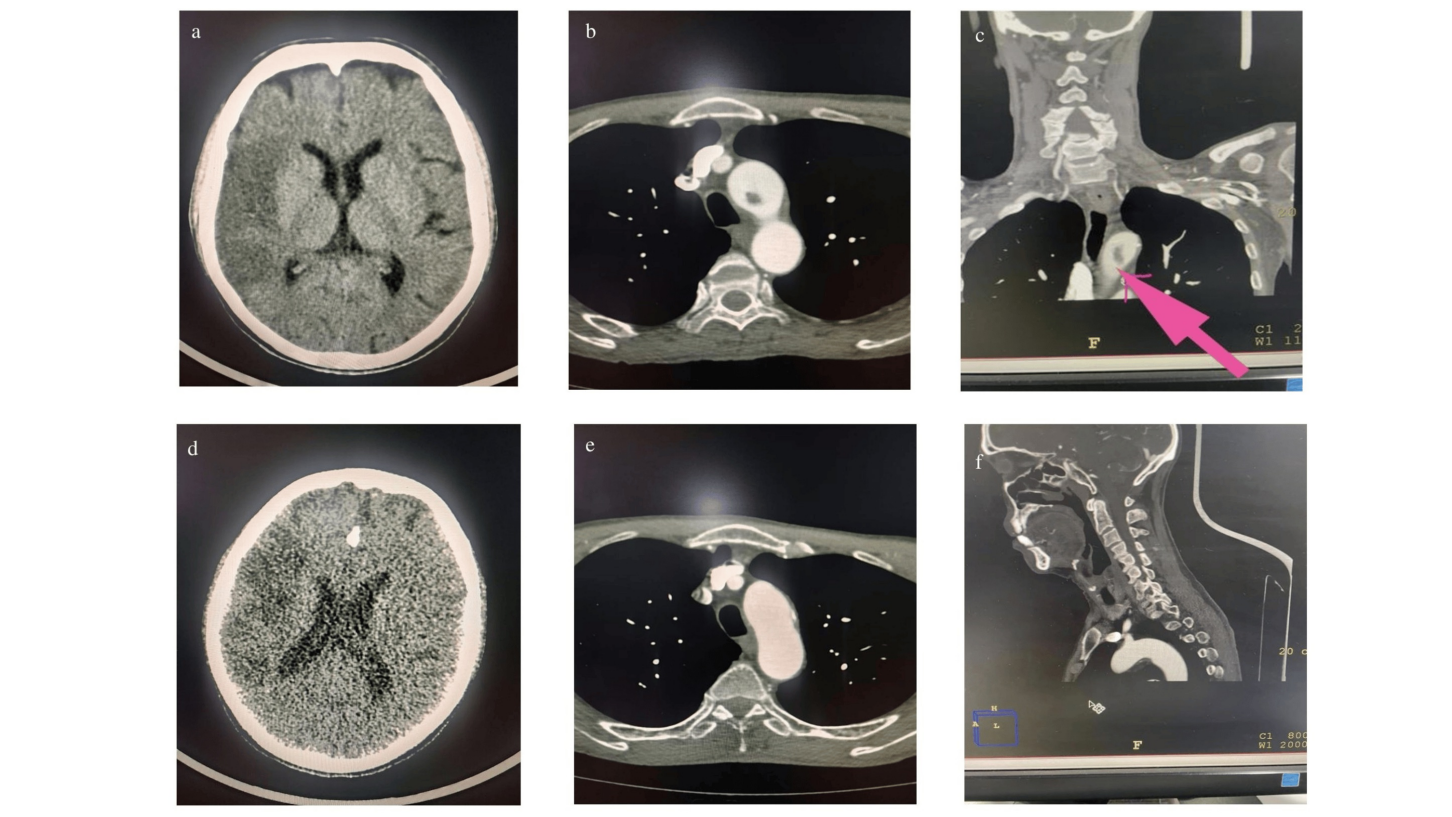
a. NCCT of the head suggestive of right fronto-temporoparietal acute infarct; b and c. Axial and coronal CT angiography sections showing a hypodense filling defect arising from the inferior wall of the aortic arch, consistent with free-floating thrombus (pink arrow); d. NCCT of the head performed on follow-up showing a near-stable infarct; e and f. Axial and coronal CT angiography sections showing no filling defect, suggestive of thrombus resolution. NCCT, non-contrast computed tomography; CT, computed tomography

A non-contrast computed tomography (NCCT) scan of the head revealed an acute infarct involving mainly the right fronto-parieto-temporal region (Figure 1a). Computed tomography angiography (CTA) of the head and neck demonstrated a hypodense filling defect at the inferior wall of the aortic arch, consistent with FFT, and an abrupt cut-off of the M1 segment of the right middle cerebral artery (MCA), suggestive of thrombotic occlusion (Figure 1b; Figure 1c). 

The patient underwent mechanical thrombectomy in the acute phase. A comprehensive etiological workup, including blood investigations, echocardiography, and Holter monitoring, was unremarkable, making an embolism secondary to an aortic arch floating thrombus the most likely source.

He was treated with single antiplatelet therapy (SAPT) (aspirin), statins, and prophylactic-dose anticoagulation (in view of the large infarcted area). The patient showed gradual neurological improvement, with motor power improving to 4/5 in both the left upper and lower limbs at discharge. On 30-day follow-up, his modified Rankin Scale (mRS) score was one, and repeat imaging demonstrated resolution of the ulcerated plaque and thrombus in the aortic arch (Figure 1e; Figure 1f).

Case 2 

A 55-year-old male, a known case of type 2 diabetes mellitus on regular medication, presented to the emergency department with sudden-onset slurring of speech; he arrived within 30 minutes of symptom onset. On examination, he had word-finding difficulty and impaired fluency, consistent with transcortical motor aphasia. The NIHSS score at presentation was 12. 

**Figure 2:**
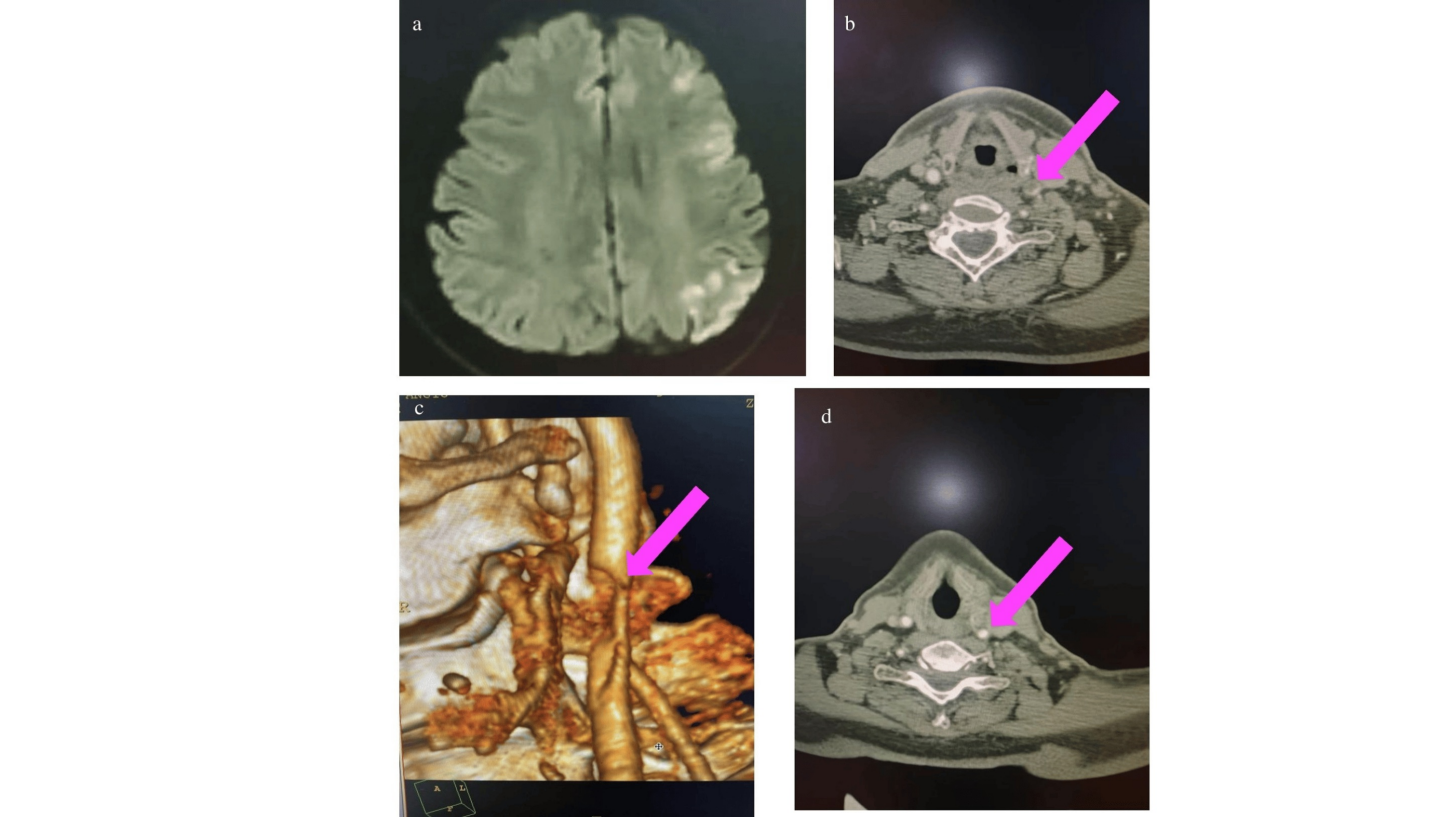
a. MRI of the brain (DWI) showing a left frontoparietal cortical region acute infarct; b and c. Axial sections of CT angiography with source imaging showing a large hypodense filling defect with an irregular surface at the left common carotid artery bifurcation, causing significant luminal narrowing, likely suggestive of a free-floating thrombus (pink arrow); d. Axial section of CT angiography showing no filling defect, suggestive of thrombus resolution (pink arrow). MRI, magnetic resonance imaging; DWI, diffusion-weighted imaging; CT, computed tomography

Magnetic resonance imaging (MRI) of the brain revealed an acute infarct involving the left frontal and parietal cortical and subcortical regions (Figure 2a). CTA of the head and neck showed a large hypodense filling defect with an irregular surface at the left common carotid artery (CCA) bifurcation, causing significant luminal narrowing, consistent with ILT (Figure 2b; Figure 2c).

As the patient was within the therapeutic window, he received IVT with alteplase. Subsequently, he was started on SAPT (aspirin) with statins, and LMWH (enoxaparin) in therapeutic doses was added in view of the identified thrombus.

A comprehensive stroke workup, including blood investigations, echocardiography, and Holter monitoring, was unremarkable, and an embolism from a carotid floating thrombus was considered the likely etiology. Follow-up CTA performed after two weeks showed a reduction in thrombus size, with only mild residual luminal narrowing (Figure 2d). The patient’s NIHSS improved to six, and he was discharged on oral anticoagulation (apixaban, therapeutic doses) in addition to statins and antiplatelet therapy.

Case 3 

A 48-year-old male with newly detected diabetes mellitus and hypertension, not yet on treatment, presented with gradually progressive right arm weakness of four days' duration. He had initially been managed for stroke at an outside facility but developed a new-onset speech disturbance on the day of presentation.

Neurological examination revealed Broca's aphasia, right-sided upper motor neuron facial palsy, and complete paralysis (0/5) of the right upper limb. The NIHSS score was 14.

**Figure 3:**
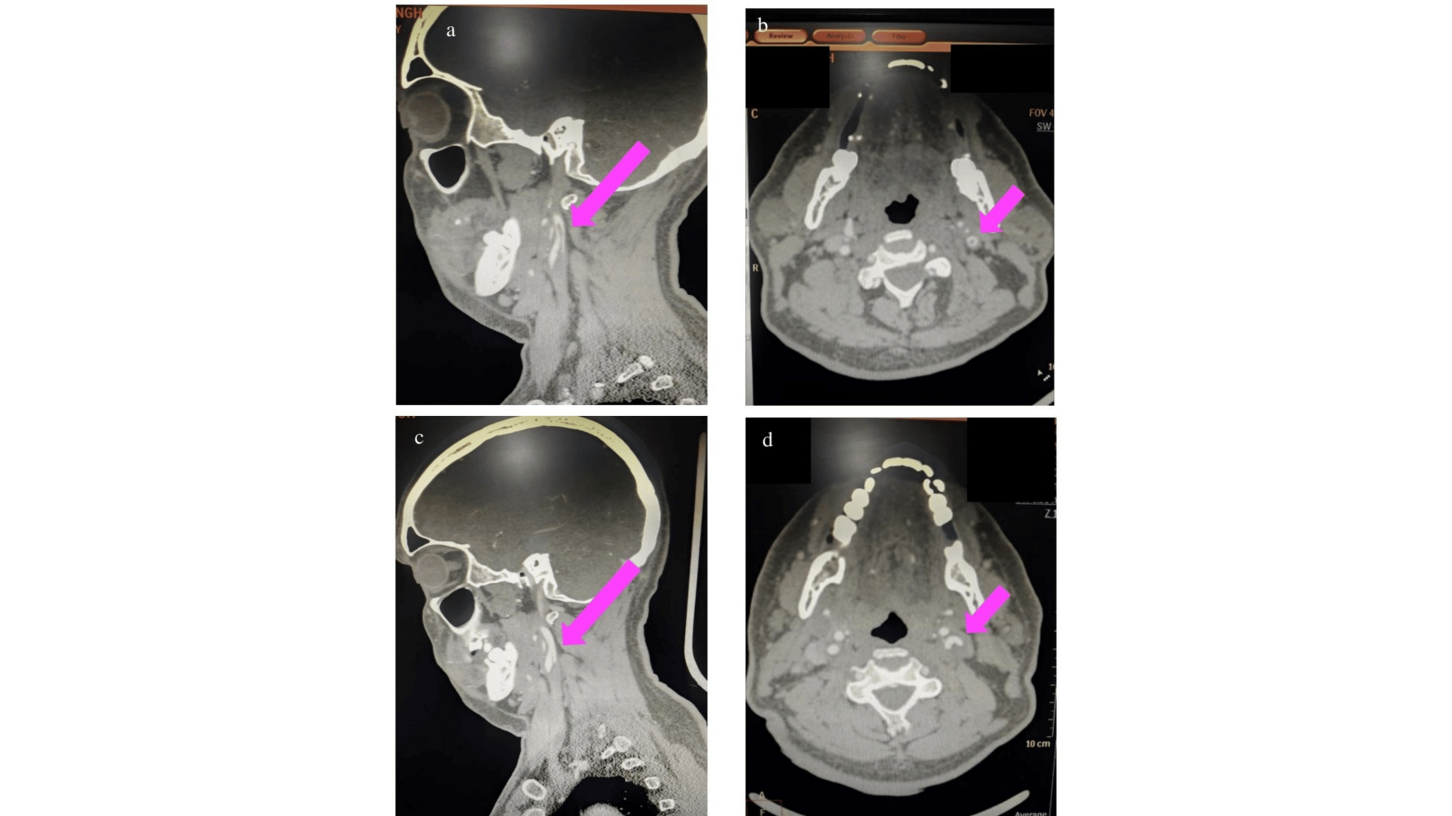
a and b. Sagittal and axial sections of CT angiography demonstrating an ulcerated atherosclerotic plaque at the left common carotid artery bifurcation, extending into the ICA, with an adjacent unstable floating thrombus (pink arrow) (a: finger sign; b: doughnut sign); c and d. Sagittal and axial sections of CT angiography showing a resolving thrombus (pink arrow). ICA, internal carotid artery; CT, computed tomography

MRI of the brain showed acute infarcts involving the left frontal, parietal, and occipital lobes. CTA demonstrated an ulcerated atherosclerotic plaque at the left CCA bifurcation, extending into the internal carotid artery (ICA), with an adjacent unstable floating thrombus (Figure 3a; Figure 3b).

He was treated with SAPT (aspirin), statins, and therapeutic anticoagulation (enoxaparin). Stroke workup, including blood tests, echocardiography, and Holter monitoring, was unremarkable, supporting an embolism secondary to carotid floating thrombus as the likely mechanism.

At follow-up, CTA performed after two weeks demonstrated a significant reduction in thrombus size (Figure 3c; Figure 3d). Clinically, the patient’s speech fluency improved, though mild word-finding difficulty persisted, and the NIHSS improved to six. He was discharged on oral anticoagulation (apixaban, therapeutic doses) and continued single antiplatelet and statin therapy.

Case 4 

A 35-year-old female with systemic lupus erythematosus (SLE) on treatment with steroids, tofacitinib, methotrexate, and hydroxychloroquine presented with sudden-onset left-hand weakness a few hours prior to arrival. Her initial NIHSS at the referring hospital was 10, and she received IVT with tenecteplase within the 4.5-hour window.

**Figure 4:**
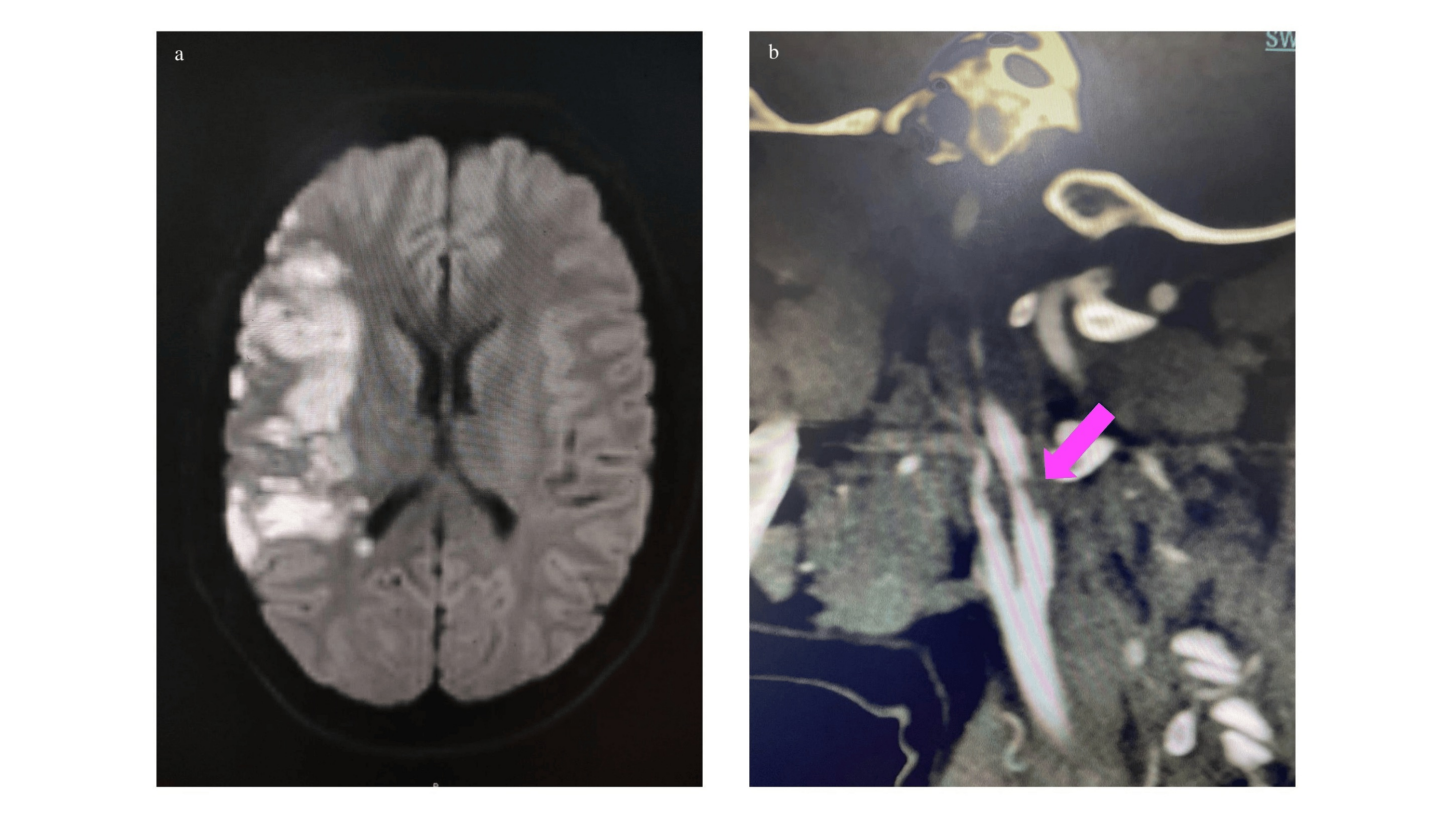
a. MRI of the brain (DWI) showing an acute infarct in the right MCA territory; b. CT angiography showing a right ICA ulcerated plaque (pink arrow). MRI, magnetic resonance imaging; DWI, diffusion-weighted imaging; MCA, middle cerebral artery; CT, computed tomography; ICA, internal carotid artery

Upon transfer and re-evaluation in our emergency department, her NIHSS had improved to four. MRI of the brain revealed an acute infarct in the right MCA territory (Figure 4a), and CTA demonstrated a right MCA inferior division occlusion with a right ICA ulcerated plaque (Figure 4b).

As the patient showed significant improvement following thrombolysis, mechanical thrombectomy was deferred. She was managed conservatively with dual antiplatelet therapy (DAPT) (aspirin and clopidogrel) and statins. Additional investigations for secondary causes of stroke were unremarkable, and a prothrombotic state secondary to SLE overlap syndrome was considered the likely etiology.

Clinically, she showed progressive improvement, with her NIHSS improving to two at discharge.

Case 5

**Figure 5:**
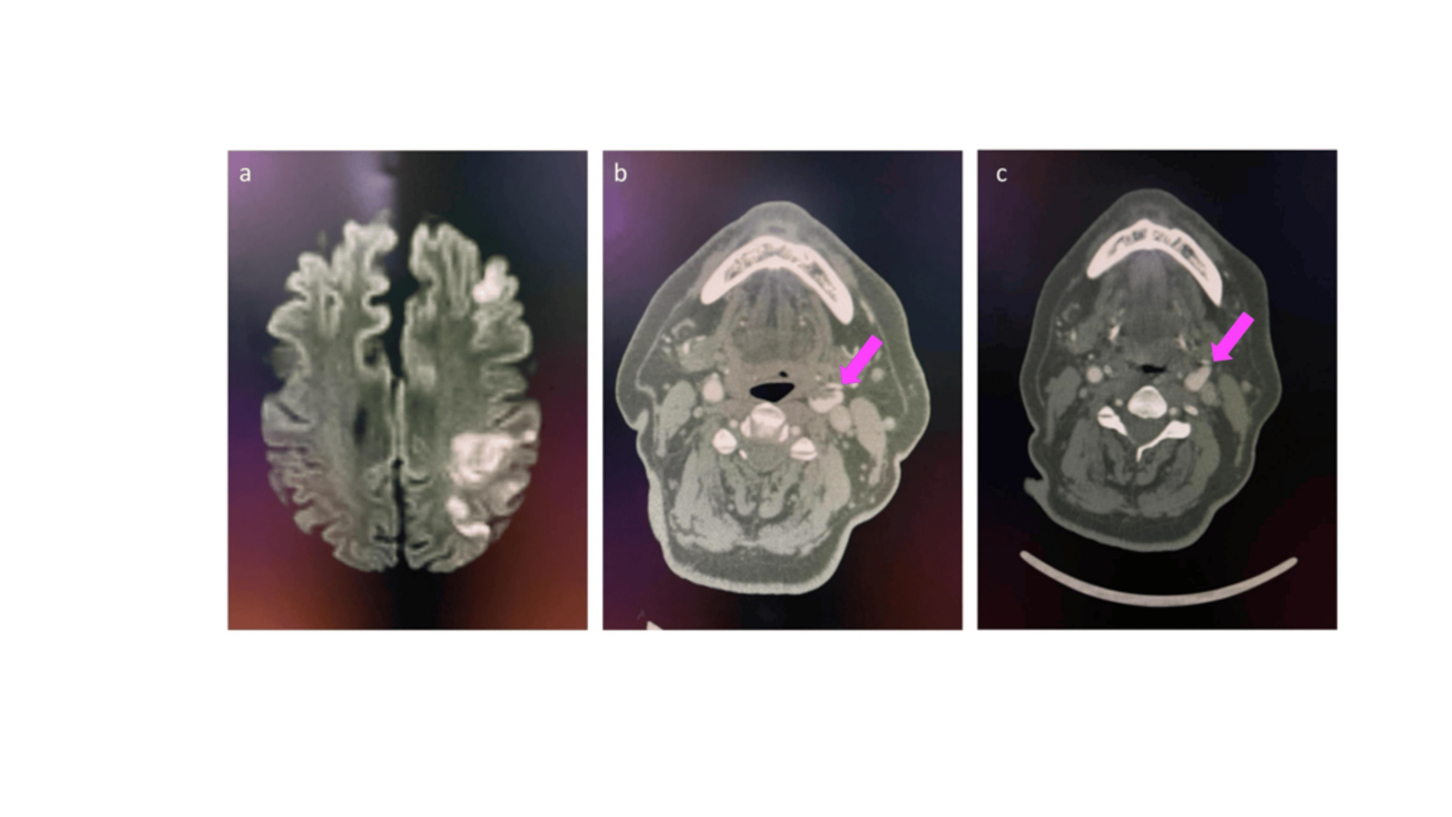
a. MRI brain DWI showing acute infarct in the left frontoparieto-occipital lobe; b. CT angiography showing left ICA free-floating thrombus extending distally (pink arrow); c. CT angiography showing complete resolution of left ICA free-floating thrombus (pink arrow). MRI, magnetic resonance imaging; DWI, diffusion-weighted imaging; CT, computed tomography; ICA, internal carotid artery

A 60-year-old male with a history of diabetes mellitus and hypertension presented to the emergency department with sudden-onset dysarthria, facial weakness, and right-sided hemiparesis 12 hours after symptom onset. AIS was suspected, and his NIHSS score at presentation was seven. MRI of the brain revealed acute infarcts involving the cortical and subcortical regions of the left fronto-parieto-occipital lobes (Figure 5a). CTA of the head and neck demonstrated an eccentric atheromatous plaque attached to the anterior wall of the left CCA bifurcation, with an unattached distal end extending into the left ICA, suggestive of FFT (Figure 5b). The patient was initiated on SAPT (aspirin and clopidogrel) and statins. A comprehensive etiological workup did not reveal any additional abnormalities, and artery-to-artery embolism from the FFT was considered the likely mechanism of stroke. On follow-up, the patient showed clinical improvement with a reduction in NIHSS score to two, and a repeat CTA performed after one month demonstrated resolution of the FFT (Figure 5c).

Table 1 summarizes each case with respect to clinical features, treatment, and follow-up.

**Table 1:** Clinical profile and outcomes of patients with ILT. mRS, modified Rankin scale; NIHSS, National Institutes of Health Stroke Scales; SLE, systemic lupus erythematosus; ICA, internal carotid artery; MCA, middle cerebral artery; ILT, intraluminal thrombus

Case	Age/sex	Risk factors/comorbidities	Thrombus location	Initial NIHSS	Treatment given	Outcome/follow-up
1	63/M	Hypertension (irregular medications), smoker	Aortic arch (inferior wall)	17	Mechanical thrombectomy, single antiplatelet (aspirin), statin, prophylactic anticoagulation → apixaban	mRS 1 and NIHSS 1; thrombus resolution
2	55/M	Diabetes mellitus	Left common carotid bifurcation	12	Intravenous thrombolysis (alteplase), single antiplatelet (aspirin), statin, therapeutic anticoagulation → apixaban	NIHSS 6; thrombus reduced
3	48/M	Newly detected diabetes mellitus, hypertension	Left carotid bifurcation extending into ICA	14	Single antiplatelet (aspirin), statin, therapeutic anticoagulation → apixaban	NIHSS 6; thrombus reduced
4	35/F	SLE (on immunosuppressants)	Right ICA ulcerated plaque with MCA occlusion	10→4 (post-thrombolysis)	Intravenous thrombolysis (tenecteplase), dual antiplatelets (aspirin and clopidogrel), statin	NIHSS 2; good recovery
5	60/M	Diabetes mellitus and hypertension	Left carotid bifurcation extending into ICA	7	Dual antiplatelets (aspirin and clopidogrel) with statins	NIHSS 2; thrombus resolution

## Discussion

Stroke is a fairly common condition caused by a thrombus in the blood vessels [5]. FFT/ILT are underdiagnosed and potentially life-threatening causes of AIS. ILT is commonly seen in the cervical arteries, such as the CCA, ICA, and vertebral artery, but can also occur in the intracranial segments and other extracervical arteries, such as the aortic arch, the brachiocephalic artery, and the subclavian artery. The prevalence of ILT among patients with AIS or transient ischemic attack (TIA) is around 3.2%, according to recent studies using CTA [6].

Consistent with previous literature, our series identified thrombi predominantly in the aortic arch and at the carotid bifurcation extending into the ICA. All five cases presented with AIS involving the anterior circulation, underscoring the high embolic potential of these lesions.

These lesions have different overlapping pathologies with differing clinical implications. The most common etiology is a complication of an atherosclerotic or ulcerated plaque. In our case series, four patients had underlying atherosclerotic risk factors, hypertension, diabetes, and smoking, suggesting plaque rupture and thrombus formation as the probable mechanism. One patient, a young female with SLE on tofacitinib, had a likely autoimmune prothrombotic mechanism. Notably, tofacitinib has also been implicated in promoting thromboembolic complications in predisposed individuals [7]. These cases highlight the heterogeneity of etiologies associated with ILT and the need for a comprehensive evaluation of vascular and systemic risk factors.

Clinically, ILTs often manifest as AIS or TIA due to distal embolization. In our series, the diagnosis was established through CTA and MRI, which not only identified the thrombus but also delineated the infarct territory and helped guide treatment decisions.

Due to the high risk of recurrent stroke and lack of evidence to direct appropriate treatment for ILT, its management is considered a therapeutic challenge and remains poorly defined. Although the best treatment strategy has yet to be determined, medical management with antithrombotics is currently the standard of care. Medical management may include SAPT, DAPT, anticoagulation, or a combination of antiplatelets and anticoagulation. Despite the agreement on antithrombotics as the medical treatment strategy in some studies, diverse antithrombotic approaches were used; however, which approach is the best remains unknown [8].

In our case series, all five patients were treated medically, using combinations of antiplatelet agents, anticoagulation (either LMWH or apixaban), and statins. Two patients received IVT (alteplase or tenecteplase) within the therapeutic window. None experienced deterioration or stroke progression, and all demonstrated clinical improvement with significant thrombus regression on follow-up imaging. These findings reinforce that medical management with antithrombotic therapy remains the mainstay of initial treatment, as also emphasized by recent reviews. The optimal duration of therapy and criteria for escalation to intervention also require further study.

Despite the high early recurrence risk reported in the literature, all patients in our cohort showed favorable outcomes, with improvement in NIHSS scores and complete or partial thrombus resolution on follow-up imaging. This favorable prognosis may be attributed to early diagnosis, prompt initiation of required therapy, and meticulous imaging follow-up.

The limitations of this case series include the small number of cases, which limits generalizability and causal inference. Additionally, this is a single-center study, and management for each patient was individualized depending on age, underlying etiology, and vessel status. Therefore, future large-scale prospective studies, especially in the Indian population, are required to clarify etiological factors and to formulate standardized management guidelines to prevent stroke recurrence and reduce the overall burden of stroke.

## Conclusions

Early recognition of FFT/ILT through high-resolution vascular imaging, such as CTA or magnetic resonance angiography, is essential for accurate diagnosis and management because of their high embolic potential and early recurrence risk. Our case series highlights the importance of prompt diagnosis and underscores the need for larger studies aimed at thrombus location characterization, underlying etiologies, and individual-level bleeding risk stratification to inform safe and effective treatment strategies. Medical management with tailored antithrombotic therapy resulted in favorable outcomes in our cases, but standardized guidelines are urgently required to optimize care.
